# Dissemination of public health information: key tools utilised by the NECOBELAC network in Europe and Latin America

**DOI:** 10.1111/j.1471-1842.2012.00977.x

**Published:** 2012-06

**Authors:** Paola De Castro, Daniela Marsili, Elisabetta Poltronieri, Carlos Agudelo Calderón

**Affiliations:** * Istituto Superiore di SanitàRome, Italy; ** Instituto de Salud Pública, Universidad Nacional de ColombiaBogotà, Colombia

**Keywords:** education and training, electronic journals, health information needs, information and communication technologies, journals, knowledge transfer, open access, publication output, publishers and publishing, research networks

## Abstract

**Background:**

Open Access (OA) to scientific information is an important step forward in communication patterns, yet we still need to reinforce OA principles to promote a cultural change of traditional publishing practices. The advantages of free access to scientific information are even more evident in public health where knowledge is directly associated with human wellbeing.

**Objectives:**

An OA ‘consolidation’ initiative in public health is presented to show how the involvement of people and institutions is fundamental to create awareness on OA and promote a cultural change. This initiative is developed within the project NEtwork of COllaboration Between Europe and Latin American Caribbean countries (NECOBELAC), financed by the European Commission.

**Methods:**

Three actions are envisaged: Capacity building through a flexible and sustainable training programme on scientific writing and OA publishing; creation of training tools based on semantic web technologies; development of a network of supporting institutions.

**Results:**

In 2010–2011, 23 training initiatives were performed involving 856 participants from 15 countries; topic maps on scientific publication and OA were produced; 195 institutions are included in the network.

**Conclusions:**

Cultural change in scientific dissemination practices is a long process requiring a flexible approach and strong commitment by all stakeholders.

**Key Messages**

**Implications for Practice**

- NEtwork of COllaboration Between Europe and Latin American Caribbean countries (NECOBELAC) supports cooperation between European and Latin American countries to make it easier to spread valuable heath information online
- Modular training material on scientific writing and open access (OA) publishing is available on the Project website to be used to ‘train the trainers’ and then for local training activities
- Course replication at local level contributes to create major awareness on health information dissemination embedding best practices at workplace

**Implications for Policy**

- NEtwork of COllaboration Between Europe and Latin American Caribbean countries networking and cooperation among European and Latin American public health institutions favours the development of shared advocacy initiatives
- Institutions and individuals from EU and LAC countries involved in the training activities are powerful tools for the adoption of institutional OA policies
- The project activity contributes to the national and international debate on scientific information dissemination in Europe and LAC, but the full evidence of its impact will be shown in the long run

## Background

Open Access to scientific information is now recognised worldwide as an important change in the information transfer process. The number of online sources is daily increasing, as well as the number of studies produced in this regard^1^ to support the transition towards a digital environment giving free access to information for all. In this framework, the development of OA journal publishing in the last 10 years proves that the value of this new publication model is currently considered as an alternative way to spread high-value scholarly information.^2^

We are now in the OA ‘consolidation’ period when we still need to reinforce the OA principles at different levels to promote a cultural change in information dissemination practices; the inclusion of new metrics for research evaluation, as an alternative to journal impact factor from Journal Citation Report (JCR), plays a relevant role in such cultural change.^3^–^5^

Within this global scenario, an increasing number of academic and research institutions utilise OA journals, repositories and other infrastructures to spread scientific information and data and are committed to foster the development of institutional, national and regional policies to support the adoption of OA publishing models.^6^

The changing conduct of science in the information age is widely discussed worldwide under different perspectives and there is a general consensus on the need of a collaborative effort to guarantee the widest use of research output at global level.^7^ The advantages of free access to information are even more evident in the field of public health where knowledge is directly associated with human well-being.^8^,^9^

This article is related to a ‘consolidation’ initiative in support of OA dissemination practices of scientific output in public health. Such initiative has been developing in Europe and Latin America within a framework of many valuable experiences promoting OA publishing, such as DRIVER (Digital Repository Infrastructure Vision for European Research), OpenAIRE (Open Access Infrastructure Research for Europe) and COAR (Confederation of Open Access Repositories) in Europe 10,11 and VHL (Virtual Health Library), SciELO (Scientific Electronic Library Online), Redalyc (Red de Revistas Científicas de América Latina y el Caribe), CLACSO (Latin American Council of Social Sciences), Lilacs (the most important and comprehensive index of scientific and technical literature of Latin America and the Caribbean) in Latin America.^12^–^14^

The European Commission is aware of the implications of OA at different levels.^15^–^17^ In this framework, the NECOBELAC project, financed by the European Commission within the 7FP- Science in Society field for the years 2009–2012, represents a consolidation initiative stressing the importance of making high-quality health information free for all.

The project NECOBELAC aims to create a network of institutions to enhance the production and dissemination of scientific information in the field of public health through a specific training strategy (http://www.necobelac.eu).^18^,^19^

The project partners are represented by institutions both in Europe and Latin America conveying different skills and experiences in information dissemination practices: Istituto Superiore di Sanità (ISS), Italy (project coordinator), Consejo Superior de Investigaciones Científicas (CSIC) Spain, the University of Nottingham (UNOTT), United Kingdom, BIREME/PAHO/WHO, Brasil, the Instituto de Salud Pública (ISP) Colombia, the Universidade do Minho (UMINHO), Portugal.

The NECOBELAC project is an example of the many collaborations supported by the European Commission within FP7 to develop a Europe-Latin America knowledge area.^20^

## Objectives

The aim of this article is to present and discuss the NECOBELAC model to meet both the global commitment of the OA movement and the local needs in the production, dissemination and use of health information. This model envisages a major involvement of local institutions and may be usefully adapted to other fields or in other geographical areas to promote a cultural change in favour of OA.

The model is applied in a wide area (European and Latin American countries) showing diverse scenarios in publishing practices associated to different socio-economic, technological and cultural factors, including language barriers, which often prevent the global circulation of valuable scientific information.^21^,^22^ The main actions included in the NECOBELAC model are described to show the results achieved so far and possible future use of the model in different contexts, namely:

### Capacity building

The aim of this action is to improve scientific writing skills of professionals working in health sciences as well as to implement scientific communication systems based on the concept of immediate, open and permanent access to research results. This should increase the publication output of institutions participating in the NECOBELAC network, improve their editorial quality and raise awareness on OA opportunities.

### Training tools

The aim of this action is to identify and realise new training tools and resources to be utilised within the capacity building programme (online and printed material, training programmes and support to local training).

### Networking

The aim of this action is the creation of a community of institutions able to promote the diffusion of health information and, at the same time, develop joint research activities.

## Methods

The methodological approach followed to meet NECOBELAC objectives is described for each action line.

### Capacity building

**Figure 1:**
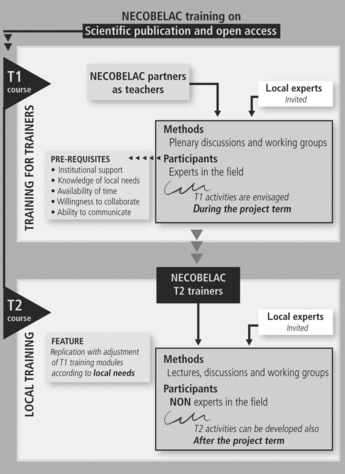
NECOBELAC two-level training strategy

It includes the development and implementation of a flexible and sustainable training programme on scientific writing and OA publishing (including repository building) for the diffusion of health information. The training strategy envisages two levels of training activities to guarantee the programme sustainability and impact (Fig. 1).^23^

- Training for trainers (*T1 activities*) on scientific publishing, including both scientific writing and OA; teachers are NECOBELAC project partners and local experts. This activity consists of eight training courses: four in Latin America and four in Europe, with an average of 30 participants (future NECOBELAC trainers) per course.
- Local training for non-experts (*T2 activities*), performed by participants in the T1 activities and assisted by project partners and local experts. The local training uses selected NECOBELAC training materials and tools (topic maps) that focus on the contextualised needs of academic and research institutions in Latin America and European countries in the concerned domain. The target has been set to have 1000 participants in the T2 activities. The methodology of course replication (T1 and T2 activities) will allow the exploitation of NECOBELAC resources and tools also beyond the NECOBELAC Project conclusion (January 2012).

Within this training programme, a questionnaire was designed to evaluate participants’ satisfaction and provide useful feedback to meet information requirements in future training activities and possibly develop new training contents. It is based on an open source software (LimeSurvey Quick statistics) and it is utilised in all T1 training courses. The questionnaire was structured into five sections (Personal information, General opinions about the course, Methodologies, Facilities and duration of the course, Suggestions to improve NECOBELAC training initiatives, Impact of the course) and allowed for multiple-choice answers.

### Training tools

Topic maps on scientific writing and OA were identified as an appropriate training tool for such a large scale project requiring great flexibility. Topic maps are based on the semantic web technology (http://code.google.com/p/ontopia/); ontopia has a navigator framework – a JSP tag library and Java API – which enables the development of web-based interfaces associated with topic maps. This technology permits the connection of relationships among different factors, actors and initiatives and represent information using ‘topics’, ‘associations’ and ‘occurrences’. The NECOBELAC topic maps consist of different modules on scientific publication and OA, each one having a scheme, a brief textual description, links to selected online resources and suggested points for discussion. This online tool was selected for its modular structure and therefore adaptability to different local training requirements.

Printed material explaining the NECOBELAC training strategy and the use of online resources (topic maps) is provided to the participants in T1 activities who commit themselves to replicate the training at local level.

### Networking

An online sample survey was planned to have an initial scenario of the areas where the project would operate. The survey was intended to collect data on scientific and academic public health institutions to be involved in the NECOBELAC capacity building programme, including data on their publication output and training activities in scientific publishing (the survey is available in four languages at http://www.necobelac.eu/Surveys/necobel.htm). The responding institutions were then invited to take part in the NECOBELAC training programme for trainers.

The network developed as a consequence of the contacts established among participants in the training activities and following the initiatives promoted for a progressive aggregation of European and Latin American institutions within the project objectives.

By supporting local training activities (T2 activities) for scientific writing and dissemination of health information, the NECOBELAC network also promotes new scientific collaborations in public health and related disciplines among institutions of the two interested geographical regions.

A discussion list and a newsletter were planned for up-dating on events, initiatives and publications related to the project themes and contribute to developing the network. Communication through social media was also envisaged.

## Results

The results of 2-year project activity are reported according to the three methodological action lines described above.

### Capacity building

Seven training courses for trainers (T1) were realised from April 2010 to August 2011. The programme of the training courses and teaching material are available online on the project website with a Creative Commons Licence 3 and therefore can be re-used without any permission (http://www.necobelac.eu/en/training.php). Table 1 shows the overall geographical distribution of NECOBELAC training activities in Europe and Latin America: 23 activities for a total of 856 attendants; 16 activities are planned in 2011 (up to August 2011).

**Table 1:** Geographical distribution of NECOBELAC training activities (2010–Aug 2011) according to type (T1, T2, planned), number and attendants

	T1*		T2**		Planned activities***
			
Geographical distribution	No. activities	No. attendants	No. activities	No. attendants	No.
Europe					
Italy	1	33	4	235	2
Portugal	1	28			1
Spain	1	19	1	50	1
United Kingdom					1
Latin America					
Argentina	1	34	4	102	2
Brazil	1	29			1
Chile			1	50	
Colombia	1	20	4	120	1
Costa Rica					
Cuba			1	50	
Ecuador					1
Mexico	1	61			4
Peru					1
Uruguay					
Venezuela			1	25	1
Total	7	224	16	632	16
Total training activities (T1+T2)	23				
Total attendants (T1+T2)	856				

* T1, Training for trainer.

** T2, Training replication activities.

*** All planned activities refer to T2 except for the United Kingdom.

Data resulting from the course evaluation questionnaire refer to five T1 courses (held in Sao Paolo, Rome, Bogotá, Madrid and Buenos Aires) of a total of seven courses held so far (August 2011). The five courses were attended by 135 people and 120 of them answered the questionnaire.

Results from the general questions which were equal for each course are reported:

- with reference to the course contents, 97% of the respondents declared that the course was very useful or useful; 98% of the respondents affirmed that the course met their expectations, definitely or somewhat; 94% of the respondents affirmed that they learned new concepts, definitely or somewhat;
- with reference to training methodology and duration of the training courses, 88% of the respondents declared that they strongly agreed or agreed with the proposed methodology;
- with reference to the teaching material, 90% of respondents declared it was adequate for their needs, definitely or somewhat;
- with reference to the duration of course (3 days), 64% declared that the length of the course was about right.

To support such positive data, there was also a rich exchange of e-mail messages from attendants soon after each course, as a mark of their involvement on the Project activities and accordingly in its community.

According to the participants’ feedback and to the evaluation of the NECOBELAC partners who played the role of teachers and facilitators in the training courses, the following considerations can be highlighted:

- the selection of participants in the training activities for trainers is crucial to guarantee replication of the training activity at local level with the support of their institutions;
- the contribution of local experts creates a major involvement at local level and helps balance local practices and priorities with international quality standards in public health information production and dissemination; in some cases, the presence of governmental authorities can help increase awareness in favour of the adoption of an OA policy to research results;
- the necessity to stimulate participation through working groups proved to be a useful tool for active learning and results in the design of feasible training programmes at local level;
- useful information on national and local practices and initiatives provided by participants in the courses helped highlight local differences and common requirements and, accordingly, adjust the project strategy for a more focussed offer of training tools and modules;
- participants in the training courses for trainers need to be supported by the project partners, operating in their geographical area, for replication of the courses at local level;
- the production of promotional printed material (leaflets, posters, bookmarks, etc.) helps disseminate information on the project, in addition to the fact that such documents are available online. Participants in the training activities also appreciate receiving NECOBELAC printed material (e.g. the ‘Guide for trainers’) to become more familiar with the project training strategy and with online resources (NECOBELAC topic maps) to use them in the replication activity.

In the T1 training course in Bogotá, active and close interaction between NECOBELAC Project partners and the course attendants led to the drawing up and signing the *Declaration of Bogotá* a position paper stating the need of sound policies promoting the quality of science communication and information process in LAC countries and outlining the commitment of the whole NECOBELAC community in this respect. The Declaration is now available in the four project languages on the Project website (http://www.necobelac.eu) and is included among the Declarations in support of OA (http://oad.simmons.edu/oadwiki/Declarations_in_support_of_OA).

### Training tools

The results achieved in this regard can be summed up into two basic groups: topic maps and support to trainers.

#### Topic maps

**Figure 2:**
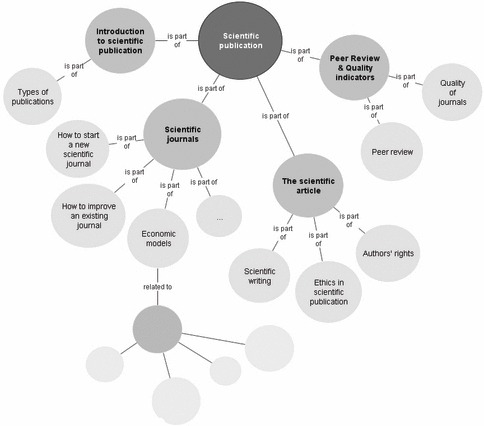
NECOBELAC topic map on scientific publication

**Figure 3:**
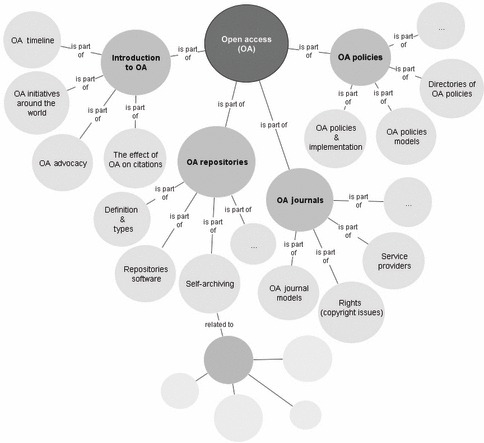
NECOBELAC topic map on open access

**Figure 4:**
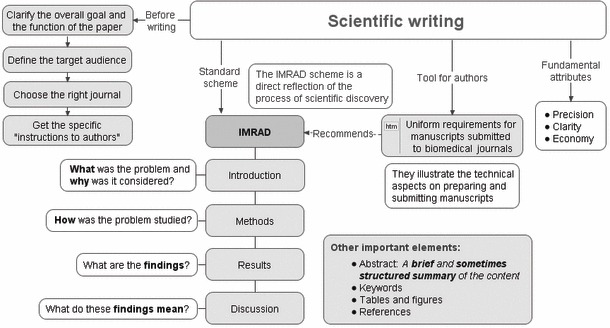
NECOBELAC scheme on scientific writing

**Figure 5:**
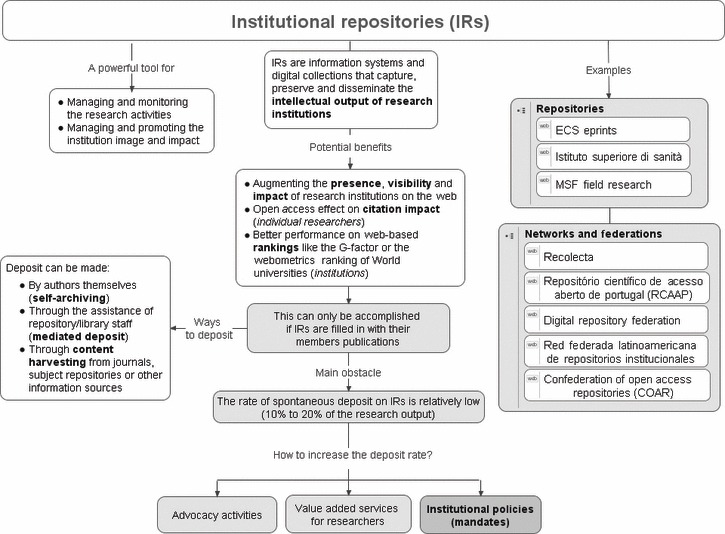
NECOBELAC scheme on institutional repositories

The development of NECOBELAC topic maps required different stages:

- knowledge representation through general and specific topics in scientific writing and OA publication; this task was also facilitated by the results of an initial online cloud-storming questionnaire utilised as a screening process for identification of terms and concepts related to those issues to determine their weight within different audience. Figures 2 and 3 display such knowledge representation showing different interconnected categories and sub-categories within the topic maps. Examples of NECOBELAC schemes for scientific publication and OA are shown in Figs 4 and 5 respectively.
- A textual description of each category and sub-category along with a list of selected links to online resources (references, Web resources) and a list of suggested questions for discussion. This is a work in progress subjected to continuous up-dating. NECOBELAC topic maps are now (August 2011) available in English and partly in Spanish, Italian and Portuguese (http://www.necobelac.eu/en/training.phpand). The publication of a printed and online book reproducing the main topics and including an introduction on the project aims and activities is also planned by the end of the project (2012).

#### Support to NECOBELAC trainers

This action has been developing through different tools:

- A guide for trainers, both online and printed, was produced to support NECOBELAC trainers in their future replication activity. The guide for trainers includes a brief description of the project strategy and the basic structure of the NECOBELAC training modules for scientific publication and OA as well as useful points for discussion and a selection of online resources.
- Constant advice is provided by NECOBELAC partners to participants in the training courses (T1) to organise local training (T2).
- A re-packaging of all training material is also envisaged to produce online training modules to be exploited in a virtual learning environment.

### Networking

The network of European and Latin American institutions involved in the project activities, starting from the initial project partner nucleus, has been continuously increasing as a result of the training initiatives and parallel actions undertaken to develop the NECOBELAC community. The initial online survey, performed from October to December 2009, at the early stage of the project, was answered by 79 institutions in Europe and LAC and was important to establish a baseline of information about the activities of institutions to be involved in the network with respect to research outputs (in terms of publications) and training courses in scientific writing and OA. The total number of institutions now included in the network is 195 (78 in Europe and 117 in Latin America); these institutions belong to 15 different countries. Detailed data are reported in Table 2.

**Table 2:** Geographical distribution of the institutions involved in NECOBELAC network (Aug 2011)

Geographical distribution	Institutions *n*
Europe	
Italy	32
Portugal	22
Spain	21
United Kingdom	3
Total Europe	78
Latin America	
Argentina	23
Brazil	32
Chile	1
Colombia	24
Costa Rica	3
Cuba	1
Ecuador	4
Mexico	22
Peru	2
Uruguay	4
Venezuela	1
Total Latin America	117
Total Europe + Latin America	195

Within the NECOBELAC network development, the bidirectional approach (EU-LAC) is focussed to stress the advantages coming from exchange of experiences and practices in health information management and use. In this framework, NECOBELAC has been supporting the major virtual library of Latin America, SciELO (Scientific Electronic Library Online, http://www.scielo.org) by suggesting European publishers of health journals to apply for inclusion in SciELO. Currently Spain and Portugal have been contributing in SciELO. Thanks to the NECOBELAC action, the science journal of the Italian National Health Institute (*Annali dell'Istituto Superiore di Sanità*) was accepted for inclusion in SciELO, in April 2010, thus representing the first Italian journal in the collection.

As a support to Europe and Latin America networking activity and to promote the publication in OA journals, NECOBELAC sustains the publication of articles in the OA journals belonging to the institutes participating in its network. Furthermore, in the beginning of 2010, the project launched an initiative to pay for publication fees of articles co-authored by European and Latin American researchers in public health and related disciplines. Although it was warmly welcomed by the NECOBELAC community, this initiative did not produce any results to date (August 2011). As an example of the low number of articles co-authored by European and Latin American researchers, a survey on *BMC Public Health* journal was undertaken to depict the scenario of scientific collaboration in public health. From a selection of papers for the years 2006, 2007 and 2008, selected according to the author affiliation, only few cases show an authorship referred to co-authors from EU and LAC countries. Only 19 of 1106 were co-authored papers, despite the great number of articles appearing per year (429 for 2008, 359 for 2007 and 314 for 2006). The majority of the articles were authored by scientists from European countries in all the years surveyed.

To develop the NECOBELAC network the project has also been establishing contacts with the other EU funded Projects within the 7th Framework Programme in the domain of health science and OA publishing, where both Latin American and European institutions are involved. The project has also been establishing positive relationships with several collaborative experiences linking Europe and Latin America, in particular, institutions from Italy, Argentina, Ecuador and Colombia collaborating on public health issues including rare diseases, medicinal products, environmental and occupational health.^24^–^27^

Within the OA global scenario, NECOBELAC shares the commitment to participate in the International OA Week (http://www.openaccessweek.org/), an annual event that is sustaining OA initiatives throughout the world. For the OA week held in 2009, NECOBELAC organised a conference in Italy at Istituto Italo-Latino Americano (IILA, http://www.iila.org) to foster EU-LAC scientific collaboration; in 2010 the project organised a training course for trainers in Italy to stress the importance of being part of an international community sharing the same objectives for OA promotion; in 2011 a series of NECOBELAC events were planned both in Europe and Latin America.

The NECOBELAC discussion list (subscriptions at: info@necobelac.eu) is very active and almost every day an up-dating on articles, news, events, documents and other relevant initiatives circulates among its members. The newsletter started in April 2011 and is now regularly issued monthly (http://www.necobelac.eu/en/newsletter.php).

A number of video interviews to NECOBELAC partners and course participants are also available on the website thus raising close involvement of people and institutions (http://www.necobelac.eu/en/VideoLink.php). Communication has also been developing through social media (Facebook, You Tube, Linkedin, etc.).

## Conclusions

The NECOBELAC project is dealing with the dissemination of scientific health information as a part of the challenging process towards a wider adoption of OA practices. Accordingly, the project aim is to contribute to a cultural change both in the production and dissemination of scientific health information. In this regard, the project is offering positive results in terms of training and networking activities thus meeting the needs to improve health information production and dissemination and contributing to capacity building at different levels.

The experience gained in the training activity carried on in Europe and Latin America, lead us to highlight the following considerations:

- the ‘core’ content of training courses for trainers must be adapted to the different local needs with high flexibility to maximise its impact. To create major involvement at local level, in each training activity local experts provided a useful contribution by reporting on local practices in journal management and OA publishing, including repository building. This favoured discussions among participants and helped providing a critical vision of the different geographical scenarios;
- group work is a basic element of training activities as it facilitates active learning and the development of friendly relationships among participants, thus promoting the organisation of joint local training activities as a required commitment from the participants in the training courses for trainers;
- the use of online resources is fundamental to empower scientific authors, editors and information specialists in respect of their different responsibility in the information transfer process, nevertheless vis à vis communication is a pre-requisite in the initial training stages;
- replication of training activity at local level has to be supported by NECOBELAC partners to ensure its realisation and therefore guarantee efficacy. Follow-up initiatives (such as communication through ad hoc mailing list, personal contacts, etc.) are required after the realisation of the courses for trainers to ensure the participants feel that they can rely on the nucleus support;
- in some cases, there is lack of knowledge of successful initiatives developed in others continents, for example, in Italy the existence of the Virtual Health Library and SciELO was generally unknown. Actions finalised to create awareness on such initiatives have to be stressed to contribute promoting new channels of information diffusion;
- the sustainability of the network requires a long-term impact strategy. In particular, the online training material must be available also after the project term to support the existing network.

Finally, we can say that NECOBELAC has been following different paths to improve and promote the exchange and sharing of information resources for the benefit of public health. Training in scientific writing and OA dissemination of research output is a way to contribute to a more equitable use of information resources worldwide and, at the same time, it is an opportunity to create new and long-standing research collaborations in public health among the European and Latin American countries participating in the project.
